# Body size dissatisfaction among young adults from the 1982 Pelotas birth cohort

**DOI:** 10.1038/ejcn.2014.146

**Published:** 2014-07-30

**Authors:** G C Mintem, B L Horta, M R Domingues, D P Gigante

**Affiliations:** 1 Postgraduate Program in Epidemiology, Federal University of Pelotas, Pelotas, Brazil; 2 Postgraduate Program in Physical Education, Federal University of Pelotas, Pelotas, Brazil

## Abstract

**Background/Objectives:**

To identify the prevalence and factors associated with body dissatisfaction.

**Subjects/Methods:**

Birth cohort study investigating 4100 subjects (2187 men and 1913 women) aged between 22 and 23 years who answered questionnaires, including the body satisfaction Stunkard Scale were included in the study; they were weighed and measured. Multinomial logistic regression was used in the crude and adjusted analyses.

**Results:**

The prevalence of body dissatisfaction was 64% (95% CI, 62.7–65.6); 42% (95% CI, 40.6–43.6) of the subjects reported feeling larger than the desired body size, and 22% (95% CI, 20.7–23.3) reported feeling smaller than desired. Underweight subjects, subjects with less schooling, poor and sedentary male subjects with low psychological well-being and female subjects who were already mothers were more likely to express body dissatisfaction, perceiving their body as smaller than the desirable body size. The prevalence of body dissatisfaction was also high among overweight subjects, subjects with a high socioeconomic status and married female subjects, who perceived their body size as too large. Minor psychiatric disorders were associated with body dissatisfaction in all subjects, regardless of perceiving themselves as larger or smaller than the desired body size. Most women perceived themselves as larger, but similar proportions of men perceived themselves as too small or too large.

**Conclusions:**

Body dissatisfaction was observed among men and women with normal weight, but it was more evident in the obese individuals. Regardless of the nutritional status, both men and women should be appropriately counseled because body size perception can lead to unhealthy behaviors in relation to diet and physical activity.

## Introduction

Body image is defined as a subjective view of one's physical appearance based on self-observation in relation to satisfaction with body size. Factors related to the perception of body image include self-esteem, well-being, acceptance in social groups, job opportunities, higher productivity, economic growth and psychosocial performance.^1, 2, 3, 4^

Body dissatisfaction may be a consequence of the increased incidence of overweight in recent decades. The prevalence and factors associated with body dissatisfaction deserve to be investigated in adulthood because of its impact on the occurrence of overweight, which is among the major public health challenges worldwide.^5^ Currently, many women desire a thin body, whereas men desire a well-defined and more muscular body shape, as advocated by the media worldwide.^6, 7, 8^ However, these goals become increasingly difficult to achieve because of eating habits and inadequate patterns of physical activity, resulting in high levels of body dissatisfaction.

Several studies on body dissatisfaction have been conducted in different countries. These studies have revealed that the prevalence of body dissatisfaction, defined as the perception that one's body size is larger than desired, is up to 90% in young adults.^4,9, 10, 11^ In a literature review about body dissatisfaction, we found only one paper that included adults from a population-based study in Brazil.^12^ In that study, more men (14%) than women (6%) exhibited body image dissatisfaction by perceiving their body as smaller than the desirable body size. Conversely, more women (67%) than men (46%) showed body image dissatisfaction by perceiving their body as larger than the desirable body size. Studies have evaluated body dissatisfaction among Brazilian men and women, specifically in young adults. All of these studies found a high prevalence of body dissatisfaction among women.^12, 13, 14, 15, 16^ The main problems in these studies were the sample size, specific population and confounding factors included in the analyses. One of these studies was a population-based study, whereas adjusted analyses using hierarchical models including socioeconomic, demographic, behavioral and health factors were conducted in two studies investigating an adult target population.

The aim of this study is to measure the prevalence of body dissatisfaction in young adults from the 1982 Pelotas birth cohort and to identify potential associated factors.

## Subjects and methods

The birth cohort study of 1982 began as a perinatal health survey of 5914 live births in the maternity hospitals of Pelotas, Brazil, to mothers who lived in the urban area of the city. Demographic, socioeconomic and health-related data were collected by a standardized questionnaire. Mothers were weighed and measured, and children were weighed. More detailed information on the study methodology has been described in previous publications.^17, 18, 19^ In the 2004–2005 follow-up, 4297 of the 5914 subjects from the birth cohort were interviewed, and all participants provided written informed consent. Physical and/or mentally disabled individuals, individuals with Down Syndrome and pregnant women, and women who had children in the 3 months prior to the interview were excluded from this analysis. As a result, 4100 subjects were included in the 2004–2005 follow-up.

The *Stunkard* Scale,^20^ with nine figures developed for each gender, was used for the outcome definition. The scale was shown along with these two questions: 'Which of these figures do you relate more with your body?' (current body size) and 'Which of these figures looks like what you would like your body to be?' (ideal body size). Differences between the chosen figures for current body size and ideal body size were classified as dissatisfaction. Positive differences indicated that the respondents perceived themselves as larger than desired, and negative differences indicated that the individuals perceived themselves as smaller than the ‘ideal'.

The association between body size dissatisfaction and demographic, socioeconomic, behavioral and health variables was investigated. Self-reported skin color, marital status (with/without a partner) and having children were included as demographic variables.

Schooling in complete years of study, family income in Brazilian currency (categorized into tertiles) and paid work in the month prior to the interview were included as socioeconomic variables.

Current smoking, physical inactivity during leisure time (less than 150 min of physical activity per week according to the International Physical Activity Questionnaire-long version)^21^ and psychological well-being (Faces Scale by Andrews, cutoffs: 1–4 for best and 5–7 for worst)^22^ were the behavioral variables.

The health variables included in the analysis were the presence of minor psychiatric disorders, as assessed by the Self Report Questionnaire-20 (cutoff point for men ⩾6 and for women ⩾8)^23^ and body mass index (BMI), calculated by the division of weight (kg) by the square of height (m^2^). Portable electronic scales (Seca Uniscale, Hamburg, Germany) with 100 *g* precision and aluminum anthropometers were used for measuring weight and height, respectively. All interviewers were trained and standardized for anthropometric measurements. Nutritional status was assessed according to the WHO criteria.^24^ BMI was classified as follows: underweight, below 18.5 kg/m^2^; adequate weight, 18.5–24.9 kg/m^2^; overweight, 25.0–29.9 kg/m^2^; and obesity, above 30 kg/m^2^.^24^

Crude and adjusted analyses using multinomial logistic regression were stratified by sex. A conceptual hierarchical framework was used to include the variables in the adjusted analysis. This conceptual model organizes the variables into hierarchical levels of determination from a more distal level to a more proximal level in relation to the outcome. In the first level, demographic and socioeconomic variables were controlled for; in the second level, behavior and health variables were controlled for and remained in the model those with *P*-value <0.2.^25^

All demographic (skin color, marital status and had children) and socioeconomic variables (education, family income and work status prior to the interview) were analyzed at the first level and remained in the analyses if the *P*-value was less than 0.2. In the second level, we included behavioral (psychological well-being, physical inactivity during leisure time and current smoking) and health variables (presence of minor psychiatric disorders and BMI). The analyses were conducted with STATA 12.0 (StataCorp, College Station, TX, USA).

## Results

**Figure 1:**
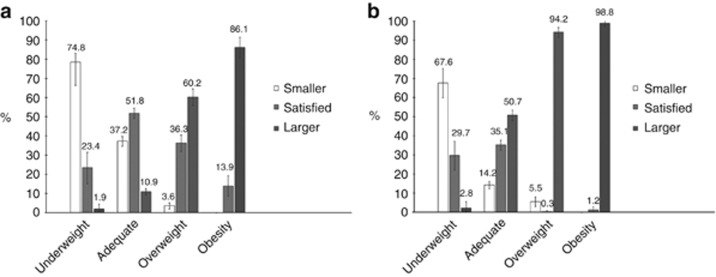
Prevalence of body image perception according to nutritional status among men (**a**) and women (**b**) in the 1982 Pelotas birth cohort aged 23 years. Pelotas –RS, 2004-5.

A total of 4100 subjects were studied, and 2187 (53%) were men. The prevalence of underweight, overweight and obesity were 6, 20 and 8%, respectively. The prevalence of body dissatisfaction was 64.1% (95% CI, 62.7–65.6). A total of 42.1% perceived themselves as larger than ideal (95% CI, 40.6–43.6), whereas 22.0% (95% CI, 20.7–23.3) considered themselves to be smaller than ideal. Among men, 27.6% (95% CI, 25.7–29.4) perceived themselves as larger than ideal and 28.5% (95% CI, 26.6–30.4) perceived themselves to be smaller than ideal, while for women, these percentages were 58.8 (95% CI, 56.5–61.0) and 14.6% (95% CI, 13.0–16.2), respectively. Overweight and obese participants perceiving themselves as larger than the ideal size showed a high prevalence of dissatisfaction. More than 90% of the overweight and obese women were dissatisfied with their body size. However, it is important to highlight the dissatisfaction in individuals classified as adequate weight; these rates were 48.1% in men and 64.9% in women (Figure 1).

Most men considered themselves to be white (skin color), had 9–11 years of formal education, had no partner (single), had no children and were currently working (Table 1). The prevalence of body dissatisfaction because of perceiving themselves as smaller than desired was higher in men who were black or mixed race; in men with lower education and income, worse psychological well-being, sedentary lifestyles, psychiatric disorders; and in men who were smokers or underweight. On the other hand, white men with higher education and income and men who were non-smokers or overweight had a higher prevalence of dissatisfaction because of perceiving themselves as larger than desired. This dissatisfaction was also more frequent among individuals with no children (Table 1).

Most women considered themselves to be white (skin color), had 9–11 years of schooling, had no partner (single), had no children (Table 2) and were currently working. The prevalence of body dissatisfaction because of perceiving themselves as smaller than desired was also higher in women with less education and lower income, worse psychological well-being, sedentary lifestyles, or psychiatric disorders and in women who were smokers or underweight. However, those who had children were more often dissatisfied because they perceived themselves as smaller. The dissatisfaction because of perceiving themselves as larger was more prevalent in women with a partner and in women who were non-smokers or overweight (Table 2).

The results of the unadjusted and adjusted multinomial analyses for men and women are presented in Tables 3 and 4, respectively. Men with less education, worse psychological well-being, sedentary lifestyles or minor psychiatric disorders and men who were underweight or in the lowest income tertile were more likely to be dissatisfied (feeling smaller than desired). Dissatisfaction because of perceiving themselves as larger remained significantly associated with schooling, income, minor psychiatric disorders and overweight (Table 3).

For women, dissatisfaction because of perceiving themselves as smaller remained significant in those who had children, less education or minor psychiatric disorders and in underweight individuals. Marital status was significantly associated with dissatisfaction among women (perceiving themselves larger), and the likelihood of dissatisfaction was higher in those with a partner. The presence of minor psychiatric disorders and overweight also remained associated with this dissatisfaction type (Table 4).

## Discussion

In Brazil, there have only been a few studies assessing body dissatisfaction in adults of both genders,^12, 13, 14, 15, 16^ which is extremely important considering the high prevalence of body dissatisfaction and its consequences to the health and nutritional status of this population. The present study identified a high prevalence of body dissatisfaction in this age group, which is understudied in our country; the majority of the studies conducted in Brazil included few subjects in the 20–30-year-old group. Dissatisfaction was observed among men and women with normal weight but was more evident in obese subjects.

According to the results of nationwide studies,^26^ the prevalence of obesity in young adults is approximately 12% in men and 17% in women; the prevalence of underweight individuals is 2% in men and 4% in women. Although the prevalence of overweight individuals is lower compared with older ages, being underweight is more common in young adults;^26^ these facts might contribute to the distortion of body image in this population.

Of the studies conducted in Brazil, three used a conceptual hierarchical model to study the variables associated with body image distortion,^12,13,16^ and two studies included demographic, socioeconomic, behavioral and health variables^12,16^ that were included in the present study.

Silva *et al.* found similar results to the present study, with the exception of the association between body dissatisfaction and socioeconomic (income and schooling) and health (minor psychiatric disorders) variables.^12^ One study including exercise facility customers examined body dissatisfaction dichotomously, which did not allow for the evaluation of the direction of the dissatisfaction.^13^ In a study of university employees assessing poor perceptions of body weight and the presence of common mental disorders, the analyses were adjusted for age and physical activity in the first conceptual model and for age, income, physical activity, self-reported health problems and BMI in the second model. In that study, only 13% of the subjects were in the 20–29-year age group.^16^

This type of analysis, which controls for potential confounders and mediators, confers greater quality to the findings of the study. This analysis could be performed in the present study, which included a larger number of variables (demographic, socioeconomic, behavioral and heath) that could influence the interpretations of our findings.

Although the *Stunkard* Scale has been widely used in epidemiological studies and validated for women in Brazil,^27^ it is still open to criticism when BMI is included in the analysis. However, studies have shown the positive aspects of this method, such as being a practical, quick and easy tool to be used in population studies, and its good correlation with BMI.^28, 29, 30^ The *Stunkard* Scale enables us to calculate discrepancies between the desired and perceived current appearance, but it is important to note that perceived discrepancy could overestimate body dissatisfaction.

The results that three out of every five men and three out of every four women were dissatisfied are important because they demonstrate a high prevalence of body dissatisfaction in young adults from a population-based study who were followed up since birth. This birth cohort has maintained a high follow-up rate, above 75% in 2004–2005.^31^ The follow-up rates did not differ according to birth weight, gender, skin color and maternal education, but losses were lower in individuals belonging to the middle levels of household income.^32^

The association between body dissatisfaction and overweight/thinness has been investigated in other studies. In a cross-sectional study with 874 university students with an average age of 20.7 years, Quadros *et al.*^14^ identified a prevalence of body dissatisfaction of approximately 78%, where 46% (34% men and 62% women) believed that they were overweight and 32% (43% men and 15% women) believed that they were too thin. In the 2008 follow-up with subjects from the 1993 Pelotas Birth Cohort, the prevalence of body dissatisfaction was 54% in boys and 61% in girls. Approximately 32% of boys wanted to be smaller and 22% wanted to be bigger, while 40% of girls wanted to be smaller and 21% wanted to be bigger.^33^

A study conducted with Brazilian female university students reported that approximately 64% of the participants desired a smaller figure.^34^ In South America, studies have found a high prevalence of body dissatisfaction in men and women, reaching values above 70%.^4,13,16,35^ It is important to note that the prevalence of dissatisfaction with body size is higher in women in most studies.

Similar results were found in studies conducted among eastern US university female students, which have found a prevalence of body dissatisfaction above 65%. In other US studies conducted with young adults, body dissatisfaction prevalence reached values close to 90%. Among men, the prevalence was lower, but also considered high, at 40–70%.^9, 10, 11^

A study carried out with young Asian adults also showed body dissatisfaction rates of approximately 60% for both women and men.^36^ This result was similar to another study conducted with Senegalese women, although the prevalences of desired larger and smaller sizes were similar.^37^

In studies conducted in the USA with young adults,^7,10^ African-American women were more likely to be dissatisfied with smaller body size. The same result was observed in the present study, as women of black or mixed skin color desired a larger body figure. However, this association did not remain after adjusting for other socioeconomic variables (education and income). These results suggest that the effects of education and income were stronger than the effect of skin color; this issue was confounded by other socioeconomic variables. The association of skin color and body dissatisfaction disappeared after controlling for demographic variables in another study performed using data from the National Physical Activity and Weight Loss Survey (NPAWLS), a population-based cross-sectional telephone survey of white, black and Hispanic US adults.^7^ However, there was no consensus regarding the effect of skin color on body dissatisfaction because the association persists after adjusting for BMI, age, marital status, and education and income levels in African-American women.^10^ Caution should be taken when interpreting these results, taking into account the race/ethnicity of the participants should be considered in the studies performed in the USA.

In the present study, men with higher education and income were more likely to perceive themselves as larger than desired. This result was still present in the adjusted analysis. It is noteworthy that the richest men were more obese and, therefore, may have perceived themselves as larger. After adjustment for BMI, the effect remained. The same was found in relation to income in the study conducted with adolescents from the 1993 Pelotas Birth Cohort,^38^ but in terms of education, opposite results were described in a study carried out with young Spaniards. In the Spanish study, a higher dissatisfaction was associated with a lower educational level.^39^ A hypothesis explaining these findings could be that young men with a higher educational level often have better economic conditions, and with muscle growth and definition, they have a way to express strength and power.

A study from all regions of Brazil showed that university students from the South perceived themselves as larger than subjects from other regions. The study also found that youths from families whose head of household had less education chose larger figures as ideal, whereas the opposite occurred in those with a head of household with more education.^34^

Lynch *et al.*^10^ showed that, among black women, higher education was associated with perceiving themselves as larger than the ideal size. The same occurred in relation to family income, although in the present study, these associations were not significant in the adjusted analysis.

With respect to BMI associations, we identified a direct association with dissatisfaction, even after adjusting for education, family income, psychological well-being, physical inactivity during leisure time, smoking and minor psychiatric disorders in men and after adjustment for marital status, having children, education, family income and minor psychiatric disorders in women. This same association was found in some studies where higher prevalence (perceiving oneself as larger than ideal) was described for males.^10,15,16,36,38,40^ For females, after adjustment, the following two variables remained associated with the outcome: marital status^41^ and, as in men, BMI.^14,16,36,42^

It should be noted that among women with adequate BMI, over half perceived themselves as dissatisfied (larger than the ideal size). A similar result was found in a cross-sectional study carried out with Chinese students and in another Brazilian study.^34,43^ This result should be emphasized considering that dissatisfaction with body image seems to be heavily influenced by the prevailing standards of beauty that advocate thinness as the ideal to be achieved and can result in unhealthy eating behaviors, causing serious health consequences.

Given the strong association between body dissatisfaction and nutritional status, the increase of overweight in both Brazilian men and women (POF 2008–2009)^26^ may be leading to a further increase in body dissatisfaction especially in those who perceive themselves as larger than the ‘ideal'. This finding should be considered worrisome for both men and women, although dissatisfaction is already quite common in women who are not overweight. Moreover, dissatisfaction because of perceiving oneself as smaller than ideal is quite frequent in healthy-weight men, which could indicate the desire for a body with greater muscle volume; however, this dissatisfaction could instead result in an increase in body fat mass.

Approximately 37% of males with adequate BMI perceived themselves as smaller than the ideal, possibly expressing a desire for a more muscular body. A similar result was found in the study conducted by Grammas *et al.*^8^ In a study conducted in low-income settings, of urban, rural Peruvian immigrants, only 43% of the study population had matching self-reported weight and BMI status, whereas 54% underestimated and 3% overestimated their BMI category.^44^

It is believed that body dissatisfaction is associated with behaviors such as smoking, physical inactivity and poor dietary habits,^45^ as well as health and psychological-related factors,^46^ including psychosomatic status,^47^ depressive symptoms^48^ and nutritional status.^10,14, 15, 16,36,38,40,42^ In this study, the presence of minor psychiatric disorders remained associated with body dissatisfaction in both directions in men and women, requiring caution when interpreting these findings because of potential reverse causality in this analysis. Thus, depression and anxiety could influence body dissatisfaction or vice-versa.

Associations between body dissatisfaction (perceiving as smaller than the ideal) and lower educational level and family income coincide with findings from other studies. Lynch *et al.*^10^ reported that men perceived themselves to be more dissatisfied as the level of education decreased, whereas among black women, only those with a lower level of education perceived themselves as smaller. In the study with adolescents from the 1993 Pelotas Birth Cohort, the lowest income was also associated with a desire to be larger for both boys and girls.^38^

According to data from POF (2008–2009), overweight was more frequent in men with higher income, whereas women presented values above 45% in all income ranges.^26^ This is a major result because, according to our findings, dissatisfaction because of perceiving oneself as larger than the ideal body size was directly associated with higher income in men and with greater BMI for both sexes.

## Conclusions

A high prevalence of body dissatisfaction in overweight subjects and in subjects with adequate nutritional status is worrisome and deserves more attention. Distinct recommendations for men and women according to socioeconomic conditions are needed, especially in relation to the income and education of this population. The high rates of body dissatisfaction found in this study suggest the need for a better understanding of the subject. In particular, it is necessary to understand how body dissatisfaction begins in young adult populations and which factors are associated with dissatisfaction in order to develop strategies that encourage the adoption of healthy lifestyles and promote a better quality of life at all stages of life.

## Figures and Tables

**Table 1:** Estimated prevalence of body dissatisfaction according to demographic, socioeconomic, environmental and health variables for men in the 1982 Birth Cohort of Pelotas. Southern Brazil, 1982 to 2004–2005

*Variables*	n	*Body image perception (%)*		
		*Smaller*	*Satisfied*	*Larger*
Skin color (*n*=2103)		*0.02*^a^		*0.03*^a^
White	1637	26.9	44.2	28.9
Black or mixed	466	33.0	44.3	22.7
Marital status (*n*=2187)		*0.91*^a^		*0.87*^a^
Without a partner	1482	28.4	44.1	27.5
With a partner	705	28.7	43.5	27.8
Had children (*n*=2187)		*0.16*^a^		*0.015*^a^
No	1666	27.7	43.4	28.9
Yes	521	30.9	45.7	23.4
Schooling (years) (*n*=2187)		*<0.001*^b^		*<0.001*^b^
⩾12	270	21.9	41.8	36.3
9–11	1005	26.9	43.6	29.5
5–8	711	30.5	45.6	23.9
0–4	201	38.3	42.3	19.4
Family income-2004–2005 (tertile) (*n*=2187)		*<0.001*^b^		*<0.001*^b^
Third	785	23.9	44.5	31.6
Second	732	27.0	43.6	29.4
First	670	35.4	43.7	20.9
Work in the month prior to the interview (*n*=2187)		*0.62*^a^		*0.35*^a^
No	490	29.4	44.7	25.9
Yes	1697	28.2	43.8	28.0
Psychological well-being (*n*=2187)		*<0.001*^a^		*0.22*^a^
Better	2060	27.6	44.5	27.9
Worse	127	43.3	33.9	22.8
Physical inactivity during leisure time (*n*=2187)		*0.02*^a^		*0.19*^a^
No	1109	26.3	47.4	26.3
Yes	1078	30.7	40.5	28.8
Smoking (*n*=2187)		*<0.001*^a^		*<0.001*^a^
No	1580	26.1	43.6	30.3
Yes	607	34.8	44.8	20.4
Minor psychiatric disorders (*n*=2183)		*<0.001*^a^		*0.61*^a^
No	1670	25.5	46.7	27.9
Yes	513	38.8	34.5	26.7
Body mass index (*n*=2184)		*<0.001*^b^		*<0.001*^b^
Underweight	107	74.8	23.4	1.9
Adequate	1410	37.2	51.8	10.9
Overweight	502	3.6	36.3	60.2
Obesity	165	0	13.9	86.1

a Chi-square test.

b Linear trend test. Italics indicate *P*-values.

**Table 2:** Estimated prevalence of body dissatisfaction according to demographic, socioeconomic, environmental and health variables for women in the 1982 Birth Cohort of Pelotas. Southern Brazil, 1982 to 2004–2005

*Variables*	n	*Body image perception (%)*		
		*Smaller*	*Satisfied*	*Larger*
Skin color (*n*=1853)		*0.06*^a^		*0.30*^a^
White	1454	14.0	27.7	58.3
Black or mixed	399	17.8	23.3	58.9
Marital status (*n*=1913)		*0.73*^a^		*0.001*^a^
Without a partner	1088	14.3	31.0	54.7
With a partner	825	14.9	21.0	64.1
Had children (*n*=1913)		*<0.001*^a^		*0.37*^a^
No	1165	11.8	30.3	57.9
Yes	748	19.0	21.0	60.0
Schooling (years) (*n*=1913)		*<0.001*^b^		*0.32*^b^
⩾12	381	8.4	29.4	62.2
9–11	981	13.0	28.4	58.6
5–8	428	21.7	20.8	57.5
0–4	123	21.1	25.2	53.7
Family income-2004-2005 (tertile) (*n*=1913)		*<0.001*^b^		*0.64*^b^
Third	606	10.4	29.5	60.1
Second	637	14.9	26.2	58.9
First	670	18.1	24.4	57.5
Work in the month prior to the interview (*n*=1913)		*0.92*^a^		*0.78*^a^
No	897	14.5	27.1	58.4
Yes	1016	14.7	26.2	59.1
Psychological well-being (*n*=1912)		*0.01*^a^		*0.45*^a^
Better	1732	13.9	27.1	59.0
Worse	180	21.7	22.2	56.1
Physical inactivity during leisure time (*n*=1913)		*0.03*^a^		*0.17*^a^
No	388	11.1	27.0	61.9
Yes	1525	15.5	26.5	58.0
Smoking (*n*=1913)		*<0.001*^a^		*0.02*^a^
No	1466	12.7	27.1	60.2
Yes	447	20.8	25.1	54.1
Minor psychiatric disorders (*n*=1911)		*0.01*^a^		*0.05*^a^
No	1286	13.1	29.7	57.2
Yes	625	17.4	20.7	61.9
Body mass index (*n*=1912)		*<0.001*^b^		*<0.001*^b^
Underweight	145	67.6	29.6	2.8
Adequate	1272	14.2	35.1	50.7
Overweight	327	0.31	5.5	94.2
Obesity	168	0	1.2	98.8

a Chi-square test.

b Linear trend test. Italics indicate *P*-values.

**Table 3:** Crude and adjusted analysis of body dissatisfaction according to demographic, socioeconomic, environmental and health variables for men in the 1982 Birth Cohort of Pelotas. Southern Brazil, 1982 to 2004–2005

*Variables*	*Smaller*		*Larger*	
	*Crude RRR (95% CI)*^a^	*Adjusted RRR (95% CI)*^a^	*Crude RRR (95% CI)*^a^	*Adjusted RRR (95% CI)*^a^
*Level 1*				
Skin color^b^ (*n*=2103)	*0.10*^c^	*0.23*^c^	*0.07*^c^	*0.30*^c^
White	1	1	1	1
Black or mixed	1.23 (0.96;1.56)	1.16 (0.91;1.49)	0.79 (0.61;1.02)	0.87 (0.66;1.13)
Marital status (*n*=2187)	*0.84*^c^		*0.82*^c^	
Without a partner	1		1	
With a partner	1.02 (0.82;1.27)		1.03 (0.83;1.28)	
Had children^b^ (*n*=2187)	*0.63*^c^		*0.04*^c^	*0.28*^c^
No	1		1	1
Yes	1.06 (0.84;1.33)		0.77 (0.60;0.99)	0.87 (0.67;1.12)
Schooling (years)^b^^d^ (*n*=2187)	*0.01*^e^	*0.04*^e^	*<0.001*^e^	*0.01*^e^
⩾12	0.58 (0.37;0.90)	0.61 (0.39;0.96)	1.89 (1.19;3.01)	1.74 (1.07;2.82)
9–11	0.68 (0.48;0.96)	0.70 (0.50;0.99)	1.47 (0.98;2.20)	1.40 (0.92;2.11)
5–8	0.74 (0.52;1.05)	0.75 (0.52;1.06)	1.14 (0.75;1.74)	1.12 (0.74;1.71)
0–4	1	1	1	1
Family income-2004–2005 (tertile)^b^^d^ (*n*=2187)	*0.001*^e^	*0.003*^e^	*0.004*^e^	*0.03*^e^
Third	0.67 (0.52;0.85)	0.78 (0.61;0.99)	1.49 (1.08;1.84)	1.39 (1.04;1.78)
Second	0.77 (0.60;0.98)	0.69 (0.54;0.88)	1.41 (1.14;1.93)	1.36 (1.06;1.83)
First	1	1	1	1
Work in the month prior to the interview (*n*=2187)	*0.88*^c^		*0.42*^c^	
No	1		1	
Yes	0.98 (0.77;1.25)		1.11 (0.86;1.41)	
				
*Level 2*				
Psychological well-being^d^^f^ (*n*=2187)	*0.001*^c^	*0.04*^c^	*0.76*^c^	
Better	1	1	1	
Worse	2.07 (1.37;3.12)	1.61 (1.03;2.52)	1.08 (0.67;1.75)	
Physical inactivity during leisure time^d^^f^ (*n*=2187)	*0.003*^c^	*0.03*^c^	*0.02*^c^	*0.05*^c^
No	1	1	1	1
Yes	1.36 (1.12;1.67)	1.27 (1.02;1.57)	1.28 (1.05;1.57)	1.28 (1.00;1.63)
Smoking^d^^f^ (*n*=2187)	*0.02*^c^	*0.47*^c^	*0.001*^c^	*0.08*^c^
No	1	1	1	1
Yes	1.30 (1.04;1.61)	1.09 (0.86;1.39)	0.66 (0.51;0.84)	0.77 (0.57;1.03)
Minor psychiatric disorders^d^^f^ (*n*=2183)	*<0.001*^c^	*<0.001*^c^	*0.04*^c^	*0.001*^c^
No	1	1	1	1
Yes	2.07 (1.63;2.61)	1.69 (1.32;2.18)	1.30 (1.01;1.67)	1.70 (1.25;2.30)
Body mass index^d^^f^ (*n*=2184)	*<0.001*^e^	*<0.001*^e^	*<0.001*^e^	*<0.001*^e^
Underweight	4.46 (2.80;7.08)	4.70 (2.90;7.62)	0.38 (0.09;1.62)	0.39 (0.09;1.67)
Adequate	1	1	1	1
Overweight	0.12 (0.07;0.20)	0.13 (0.08;0.21)	7.88 (6.11;10.15)	8.19 (6.32;10.61)
Obesity			29.31 (18.25;47.06)	30.57 (18.89;49.46)

Abbreviation: RRR, relative risk rates.

a RRR satisfied was used as base outcome.

b Variables adjusted to each other.

c Test for heterogeneity.

d Adjusted analysis according a conceptual hierarchical model and remained in the adjusted model variables with *P*-value<0.20.

e Test for Linear trend.

f Adjusted for level 1 variables and to each other. Italics indicate *P*-values.

**Table 4:** Crude and adjusted analysis of body dissatisfaction according to demographic, socioeconomic, environmental and health variables for women in the 1982 Birth Cohort of Pelotas. Southern Brazil, 1982 to 2004–2005

*Variables*	*Smaller*		*Larger*	
	*Crude RRR (95% CI)*^a^	*Adjusted RRR (95% CI)*^a^	*Crude RRR (95% CI)*^a^	*Adjusted RRR (95% CI)*^a^
*Level 1*				
Skin color^b^ (*n*=1853)	*0.02*^c^	*0.26*^c^	*0.18*^c^	*0.23*^c^
White	1	1	1	1
Black or mixed	1.52 (1.07;2.15)	1.23 (0.85;1.78)	1.20 (0.92;1.57)	1.18 (0.90;1.55)
Marital status^b^^d^ (*n*=1913)	*0.01*^c^	*0.98*^c^	*<0.001*^c^	*<0.001*^c^
Without a partner	1	1	1	1
With a partner	1.54 (1.14;2.07)	1.01 (0.71;1.42)	1.73 (1.39;2.15)	1.61 (1.26;2.05)
Had children^b^^d^ (*n*=1913)	*<0.001*^c^	*0.004*^c^	*<0.001*^c^	*0.22*^c^
No	1	1	1	1
Yes	2.33 (1.73;3.15)	1.72 (1.19;2.49)	1.50 (1.20;1.87)	1.17 (0.91;1.51)
Schooling (years)^b^^d^ (*n*=1913)	*<0.001*^e^	*0.01*^e^	*0.25*^e^	
⩾12	0.34 (0.18;0.65)	0.52 (0.26;1.07)	0.99 (0.61;1.61)	
9–11	0.55 (0.31;0.96)	0.72 (0.40;1.30)	0.97 (0.62;1.52)	
5–8	1.25 (0.69;2.26)	1.31 (0.72;2.38)	1.30 (0.79;2.12)	
0–4	1	1	1	
Family income 2004-5 (tertile)^b^^d^ (*n*=1913)	*<0.001*^e^	*0.05*^e^	*0.27*^e^	
Thirrd	0.48 (0.33;0.69)	0.66 (0.44;0.99)	0.87 (0.67;1.12)	
Second	0.77 (0.55;1.09)	0.97 (0.68;1.40)	0.96 (0.74;1.24)	
First	1	1	1	
Work in the month prior to the interview (*n*=1913)	*0.78*^c^		*0.70*^c^	
No	1		1	
Yes	1.05 (0.78;1.40)		1.04 (0.86;1.29)	
				
*Level 2*				
Psychological well-being^f^ (*n*=1912)	*0.01*^c^	*0.91*^c^	*0.44*^c^	
Better	1	1	1	
Worse	1.90 (1.20;3.05)	0.97 (0.56;1.67)	1.16 (0.79;1.70)	
Physical inactivity during leisure time^f^ (*n*=1913)	*0.08*^c^	*0.33*^c^	*0.73*^c^	
No	1	1	1	
Yes	1.42 (0.96;2.10)	1.24 (0.80;1.92)	0.95 (0.74;1.24)	
Smoking^f^ (*n*=1913)	*0.001*^c^	*0.22*^c^	*0.85*^c^	
No	1	1	1	
Yes	1.78 (1.28;2.46)	1.27 (0.87;1.85)	0.98 (0.76;1.26)	
Minor psychiatric disorders^d^^f^ (*n*=1911)	*<0.001*^c^	*0.003*^c^	*<0.001*^c^	*<0.001*^c^
No	1	1	1	1
Yes	1.93 (1.41;2.64)	1.73 (1.21;2.47)	1.56 (1.23;1.97)	1.46 (1.13;1.88)
Body mass index^d^^f^ (*n*=1912)	*<0.001*^e^	*<0.001*^e^	*<0.001*^e^	*<0.001*^e^
Underweight	5.66 (3.80;8.43)	7.15 (4.66;10.98)	0.06 (0.02;0.18)	0.07 (0.02;0.19)
Adequate	1	1	1	1
Overweight	0.12 (0.02;0.93)	0.11 (0.01;0.81)	11.86 (7.26;19.36)	11.24 (6.87;18.38)
Obesity			57.51 (14.19;233.08)	54.34 (13.40;230.39)

Abbreviation: RRR, relative risk rates.

a RRR satisfied was used as base outcome.

b Variables adjusted to each other.

c Test for heterogeneity.

d Adjusted analysis according a conceptual hierarchical model and remained in the adjusted model variables with *P*-value<0.20.

e Test for Linear trend.

f Adjusted for level 1 variables and to each other. Italics indicate *P*-values.
